# Urinary Screening for Aminoacidurias Using Chromatography and Serum Amino Acid Profile in Type 2 Diabetes and Healthy Controls

**DOI:** 10.1155/bri/4060832

**Published:** 2025-03-17

**Authors:** Sushma B. J., Sumit Parashar, Balvir Singh Tomar, Alka Meena, Priyanka B. J.

**Affiliations:** ^1^Department of Biochemistry, National Institute of Medical Sciences and Research, NIMS University, Jaipur, Rajasthan, India; ^2^Institute of Paediatric Gastroenterology and Hepatology, NIMS University, Jaipur, Rajasthan, India; ^3^Department of Biochemistry, SMS Medical College, Jaipur, Rajasthan, India; ^4^Department of CSE, NIMS Institute of Engineering Technology, NIMS University, Jaipur, Rajasthan, India

**Keywords:** amino acids, aminoacidurias, gas chromatography–tandem mass spectrometry, paper chromatography, type 2 diabetes mellitus

## Abstract

Type 2 diabetes is a complex metabolic disorder characterised by altered amino acid metabolism. This study investigates plasma amino acid profiles and specific aminoacidurias in patients with type 2 diabetes. We recruited 115 patients with type 2 diabetes and 115 healthy controls. Urine amino acids were analysed using paper chromatography, while serum amino acids were analysed using gas chromatography–mass spectrometry (GC-MS). Diabetes-induced alterations in amino acid metabolism are multifaceted. Hyperglycemia and insulin resistance can lead to increased gluconeogenesis, resulting in the depletion of certain amino acids, such as glutamine and arginine. Conversely, the increased availability of branched-chain amino acids (BCAAs) such as leucine, isoleucine and valine can contribute to insulin resistance and impaired glucose metabolism. Significant alterations in plasma and urine amino acid levels were observed in patients with type 2 diabetes, including increased levels of BCAAs and decreased levels of glutamine and arginine. These changes correlated with disease severity and metabolic parameters. Understanding these alterations is crucial for developing novel therapeutic strategies and diagnostic tools for type 2 diabetes management. Our findings provide new insights into amino acid metabolism in type 2 diabetes, identifying potential biomarkers for disease diagnosis and monitoring. These results suggest avenues for developing therapeutic strategies and contribute to understanding the complex relationship between amino acid metabolism and type 2 diabetes. The study highlights the potential of amino acid profiling as a diagnostic and therapeutic tool in type 2 diabetes management.

## 1. Introduction

Type 2 diabetes mellitus (T2DM) has emerged as a critical global health concern, currently affecting over 460 million people worldwide. This number is projected to rise to 700 million by 2030, with a disproportionate impact on low- and middle-income countries [1]. Factors such as sedentary lifestyles, unhealthy diets, obesity and genetic predisposition contribute significantly to the increasing prevalence of type 2 diabetes (T2DM). The disease also significantly elevates the risk of severe complications such as cardiovascular disease, kidney failure and other debilitating conditions. As the world grapples with this epidemic, understanding the complex pathophysiology of T2DM, along with identifying effective prevention and treatment strategies, is of utmost importance [2].

Specific amino acids play an essential role in maintaining glucose homeostasis and regulating insulin secretion from pancreatic β-cells. Diabetes-induced alterations in amino acid metabolism—such as disruptions in the synthesis, transport and utilisation of specific amino acids—are strongly associated with insulin resistance and the progression of T2DM. One of the key manifestations of these metabolic disturbances is aminoaciduria, which refers to the excessive excretion of amino acids in the urine. This phenomenon underscores the complex interplay between glucose and amino acid homeostasis, which can exacerbate insulin resistance and glucose intolerance [3, 4].

In T2DM, diabetes-induced alterations in renal function, such as impaired reabsorption and changes in amino acid transporter activity, contribute to the development of aminoaciduria. These alterations compromise protein synthesis and tissue repair processes, further complicating the metabolic dysfunction associated with the disease. By elucidating the metabolic significance of these diabetes-induced alterations, particularly in the context of aminoaciduria, we may uncover novel therapeutic targets for addressing protein-energy wasting and improving glycaemic control in diabetic patients [5–7].

The primary objectives of this study were as follows: (a) to investigate and compare the frequency of specific aminoacidurias in patients with T2D and healthy controls, (b) to analyse and compare the serum amino acid profiles between T2D patients and healthy controls and (c) to assess the correlation between the degree of aminoaciduria and glycaemic control (as measured by HbA1c) in T2D patients.

## 2. Materials and Methods

### 2.1. Study Design and Study Settings

This cross-sectional study included 115 type 2 diabetic subjects, who were on oral anti-diabetic medication with duration less than 10 years from the time of diagnosis, diagnosed as per ADA criteria and 115 healthy controls in the age group of 25–65 years of both genders. The sample size was calculated using the formula *n*=(Z_α/2_+Z1_−β_) 2 × (*σ*_1_^2^+*σ*_2_)/(▲)^2^ using 95% confidence interval. The study was conducted in the Department of Biochemistry in association with the Department of General Medicine at the National Institute of Medical College and Research after obtaining approval from the Institutional Ethical Committee, NIMS University, Jaipur. Informed written consent was obtained from all the subjects prior to the study. We excluded the patients with type 1 diabetes, type 2 diabetic subjects on insulin treatment, urinary tract infections, renal failure, diabetic nephropathy, renal glycosuria and known cases of inborn errors of metabolism.

### 2.2. Sample Collection and Biochemical Analysis

Whole Blood and Plasma: under aseptic precautions, 2 mL of venous blood sample was collected in the EDTA vial after overnight fasting and used for estimation of glycated haemoglobin (HbA1c) by the HPLC method, and the sample is subjected for centrifugation for the separation of plasma used for fasting blood glucose estimation by glucose oxidase–peroxidase method (GOD-POD method) in fully automated integrated biochemistry analyser (Vitros 5600). All the serum samples were stored at −80°C until analyses were performed for serum amino acids. Specific amino acids levels in the serum were measured by using gas chromatography–tandem mass spectrometry as per the manufactures' instructions [8].

### 2.3. Paper Chromatography (PC)

PC is a versatile, inexpensive noninvasive analytical technique that separates and identifies amino acids and other biomolecules based on their affinity for a stationary phase and mobility in a mobile phase, which is calculated as a retention factor (Rf).

PC was standardised in our central research biochemistry laboratory using standard amino acids. The reagents required for the procedure were as follows: (1) the solvent was prepared in the ratio of 12:3:5 using butanol: acetic acid: water. (2) Staining amino acids: 0.1% ninhydrin solution in acetone. All 18 readymade standard acid solutions were applied separately as 4 μL spots on chromatography paper and were allowed to dry, and the paper was dipped in the chromatography chamber containing the solvent, which was allowed the solvent to migrate 10–15 cm, typically 2–4 h.

The paper was air-dried at ambient temperature for 30 min. A 0.1% ninhydrin solution was uniformly sprayed onto the paper, ensuring consistent coverage across replicate amino acid spots and avoiding excessive moisture accumulation. Following spraying, the paper was allowed to stand at room temperature for 30 min before being subjected to a precise 2-min heat treatment at 100°C. The Rf values for each amino acid were subsequently calculated using the following formula: Rf = distance travelled by solute/distance travelled by solvent. This paper served as the reference standard for Rf values. Early morning first voided mid-stream urine sample of 5 mL was collected from both type 2 diabetic subjects and healthy controls into the urine container and subjected to centrifugation to remove cell debris, and the clear supernatant was used for PC. Urine samples from type 2 diabetic subjects and controls were processed and applied as 4 μL spots on chromatography paper. The paper was then immersed in a solvent system comprising butanol, acetic acid, and water and allowed to develop via chromatography. Subsequent identification of amino acids in the urine samples was achieved by comparing their Rf values with those obtained from the standard reference paper.

### 2.4. Ethical Consideration and Consent

The study was conducted after taking the Institutional Ethical Committee clearance with the Reference No EC/New/Inst/2022/RJ/0118 and Proposal Number IEC/P 427/2023. Informed written consent was obtained from all the subjects (type 2 diabetic subjects and healthy controls) involved in the study. All methods were performed in accordance with the relevant guidelines and regulations.

## 3. Results and Discussion

Aminoaciduria, characterised by excessive amino acid excretion, is a frequent complication in T2D patients, reflecting impaired renal reabsorption and altered metabolic pathways. The elevated urinary loss of essential amino acids, particularly branched-chain amino acids (BCAAs), may exacerbate insulin resistance and glucose dysregulation. Studies suggest that aminoaciduria in T2D is linked to increased oxidative stress, inflammation and renal damage. Moreover, certain amino acid imbalances, such as elevated glutamine and decreased glycine, may contribute to the development of diabetic nephropathy. Early detection and management of aminoaciduria may provide a valuable adjunctive strategy for mitigating T2D-related complications.

In the present study, 115 healthy controls and 115 type 2 diabetic subjects were enrolled as per the inclusion and exclusion criteria. The age-wise distribution of the subjects indicated that most of the type 2 diabetic subjects were in the age group of 50–60 years as compared to the controls, as presented in Table 1.

The gender-wise distribution of the study subjects shows that the male subjects were higher than female subjects, as indicated in Table 2.

Table 3 represents the baseline parameters' comparison; it is found that the fasting blood glucose, systolic blood pressure, diastolic blood pressure and HbA1c values were elevated (*p* < 0.001) in type 2 diabetic subjects in comparison with healthy controls, and type 2 diabetic subjects were older (*p* < 0.001) compared to healthy controls.

The overall frequency of specific aminoacidurias was higher in type 2 diabetic subjects as compared to healthy controls. Specific aminoacidurias of glutamic acid (58.26% vs. 9.57%), valine (34.78% vs. 4.35%), lysine (16.52% vs. 9.57%), leucine (11.30% vs. 1.74%), alanine (23.48% vs. 2.61%), serine (25.22% vs. 2.61%), tryptophan (10.43% vs. 0.87%), phenylalanine (10.43% vs. 1.72%), glycine (10.43% vs. 2.61%), histidine (20% vs. 3.48%), tyrosine (6.09% vs. 0%), proline (10.43% vs. 1.74%), cystine (11.3% vs. 4.35%) and arginine (6.96% vs. 1.74%) showed statistically high significance in T2D as compared to healthy controls. Aminoacidurias of isoleucine, aspartic acid, threonine and methionine did not show statistical significance, even though the prevalence was higher in type 2 diabetic subjects; the data are represented in Table 4.

The mean Rf values were compared between the two groups; it is found that the Rf values of PC was statistically significantly higher in type diabetic subjects as compared to healthy controls (*p* < 0.001), as depicted in Table 5.

Table 6 presents the plasma concentrations of specific amino acids analysed in the serum. The levels of certain amino acids, glutamic acid, valine, lysine, leucine, isoleucine, tyrosine, threonine and arginine were elevated, and glycine, alanine and histidine levels were decreased in type 2 diabetic subjects as compared to healthy controls (*p* < 0.001). The concentration of certain amino acids such as serine, tryptophan, phenylalanine, aspartic acid, methionine, proline and cystine did not show statistical significance (*p* > 0.05).

BCAAs, comprising leucine, isoleucine and valine, are essential amino acids that play a vital role in glucose metabolism and insulin sensitivity. The mechanistic target of the rapamycin (mTOR) pathway is a critical regulator of cellular growth, metabolism and autophagy. BCAAs, particularly leucine, activate the mTOR pathway, leading to increased insulin resistance by reducing insulin receptor substrate-1 (IRS-1) expression, enhanced glucose production in the liver by stimulating gluconeogenesis and impaired glucose uptake in the skeletal muscle [9–11]. In the present study, we found elevated serum levels of BCAA and increased urinary excretion in T2D (*p* < 0.001); this was in accordance with Kolanu et al. [12]. Another study conducted by Bidi et al. compared the urinary excretory pattern of amino acids in T2D and healthy controls, and the study showed a higher frequency of excretion of amino acids phenyl alanine, arginine, tyrosine and tryptophan [13, 14]. The present study shows increased excretion of all the 18 amino acids, out of which 14 amino acids excretion was statistically significant (*p* < 0.001).

Table 7 represents the correlation between glycaemic control and degree of amino acidurias (number of urine amino acids detected by PC). The degree of aminoacidurias was significantly positively correlating with the increased HbA1c levels indicating the direct relationship between glycaemic control and impaired amino acid reabsorption. This is the first study to establish the relationship between the degree of aminoaciduria and glycaemic control in T2DM.

A study conducted in Chinese population with diabetic kidney disease (DKD) found significant reduction in the plasma levels of histidine as compared to diabetic subjects without DKD. This indicate the role of these amino acids as diagnostic biomarkers to identify the renal impairment in T2DM [15]. The research has showed that insufficient levels of histidine in the blood can exacerbate inflammation and oxidative stress in kidney diseases. Fortunately, studies have found that supplementing the diet with histidine can help alleviate these issues. The underlying cause of abnormal histidine levels is thought to be related to imbalances in histidine metabolism. Maintaining healthy histidine levels is essential for preventing complications associated with kidney diseases [16, 17].

Pharmacological interventions, in addition to lifestyle modifications, have shown promise in preventing or delaying the onset of T2DM. Tailored pharmacological approaches can potentially mitigate the detrimental effects of amino acid imbalance on cellular signalling pathways. Amino acid metabolic pathways may emerge as key targets for pharmacological therapies [18]. Prolonged metformin treatment can modulate circulating BCAA levels, and its effects extend beyond activating AMPK and reducing hepatic gluconeogenesis. Metformin also downregulates the expression of BCAA catabolic enzymes, such as BCAT2 and BCKDHa. In addition, the combination therapy with glipizide and metformin has been found to acutely alter levels of BCAAs and aromatic amino acids, reflecting improved glyacemic control. Low-dose metformin treatment has also shown to rectify glucose metabolic imbalances, with integrative metabolomics analysis revealing changes in amino acid levels, including increases in serine, glycine and glutamate, and decreases in aspartate [19–22]. Furthermore, SGLT2 inhibitors such as empagliflozin have been found to increase concentrations of BCAA metabolites, while DPP4 inhibitors such as sitagliptin have shown to decrease plasma valine levels and alter amino acid patterns in both mice and patients with T2D [23]. Further research is needed to elucidate the mechanisms underlying these complex associations and to develop novel, effective pharmacologic therapies for T2D.

## 4. Conclusion

Assessing plasma and urine amino acid profiles is crucial for understanding T2D pathophysiology. These profiles can help identify individuals at risk, monitor disease progression, and evaluate treatment response. Targeting amino acid metabolism may offer new therapeutic approaches for managing T2D. Integrating amino acid analysis into clinical practice can improve patient outcomes.

## Figures and Tables

**Table 1:** Frequency distribution of age of healthy controls and type 2 diabetic subjects.

Age interval	Controls		Diabetes mellitus	
	*n* = 115	In (%)	*n* = 115	In (%)
≤ 30	60	52.17	10	8.70
31–40	17	14.78	13	11.30
41–50	13	11.30	29	25.22
51–60	25	21.74	63	54.78

**Table 2:** Frequency distribution of gender of healthy controls and type 2 diabetic subjects.

Gender	Controls		Diabetes mellitus	
	*n* = 115	In (%)	*n* = 115	In (%)
Male	89	77.39	68	59.13
Female	26	22.61	47	40.87

**Table 3:** Comparison of baseline parameters between healthy controls and type 2 diabetic subjects using *t*-test.

Variables	Controls	Diabetes mellitus	*t*-test	*p* value	Significance
Age	34.77 ± 13.73	49.1 ± 11.13	−8.699	0.00001	Significant
Systolic blood pressure	118.29 ± 8.68	134.6 ± 16.54	−9.364	0.00001	
Diastolic blood pressure	80.82 ± 6.36	87.76 ± 6.02	−8.499	0.00001	
Fasting blood glucose	89.36 ± 19.92	190.8 ± 143.34	−7.520	0.00001	

Serum creatinine	0.71 ± 0.22	0.83 ± 0.69	−1.799	0.07335	Not significant

HbA1c	5.28 ± 0.29	8.93 ± 2.15	−18.02	0.00001	Significant

**Table 4:** Comparing specific aminoacidurias between healthy controls and type 2 diabetic subjects' group by using normal *Z*-test.

Amino acids	Controls (%)	Diabetes mellitus (%)	*Z*-test	*p* value	Significance
Glutamic acid	9.57	58.26	−9.095	0.00001	Significant
Valine	4.35	34.78	−6.299	0.00001	
Lysine	6.09	16.52	−2.533	0.00565	
Leucine	1.74	11.30	−2.994	0.00138	
Alanine	2.61	23.48	−4.942	0.00001	

Isoleucine	0.87	1.74	−0.582	0.28042	Not significant

Serine	2.61	25.22	−5.241	0.00001	Significant
Tryptophan	0.87	10.43	−3.211	0.00066	
Phenylalanine	1.74	10.43	−2.805	0.00252	
Glycine	2.61	10.43	−2.434	0.00746	
Histidine	3.48	20.00	−4.027	0.00003	
Tyrosine	0.00	6.09	−2.730	0.00317	

Aspartic acid	2.61	6.09	−1.298	0.09713	Not significant
Threonine	0.00	1.74	−1.427	0.07684	
Methionine	1.74	4.35	−1.155	0.12407	

Proline	1.74	10.43	−2.805	0.00252	Significant

Cystine	4.35	11.30	−1.981	0.02381	Significant

Arginine	1.74	6.96	−1.956	0.02523	Significant

**Table 5:** Comparing the mean Rf values of paper chromatography in healthy controls and type 2 diabetic subjects.

Amino acids	Controls	Diabetes mellitus	*Z*-test	*p* value
Rf value	0.142 ± 0.066	0.312 ± 0.062	−3.095	0.00001

**Table 6:** Comparing the serum levels of specific amino acids between healthy controls and type 2 diabetic subjects.

Amino acids (micromole/L)	Controls	Diabetes mellitus	Significance
Glutamic acid	164.86 ± 12.64	226.54 ± 10.96	Significant
Valine	200.32 ± 8.65	332.43 ± 12.82	Significant
Lysine	128.32 ± 4.98	232.42 ± 5.99	Significant
Leucine	48.43 ± 5.73	64.56 ± 7.24	Significant
Alanine	24.64 ± 8.54	18.64 ± 5.42	Significant
Isoleucine	88.64 ± 3.89	186.32 ± 6.98	Significant
Serine	96.42 ± 6.32	94.26 ± 8.24	Not significant
Tryptophan	62.43 ± 8.24	66.45 ± 9.26	Not significant
Phenylalanine	88.98 ± 10.24	92.43 ± 6.56	Not significant
Glycine	360.64 ± 32.86	198.86 ± 28.43	Significant
Histidine	88.42 ± 3.98	72.64 ± 2.64	Significant
Tyrosine	124.56 ± 10.26	164.56 ± 10.261	Significant
Aspartic acid	198.32 ± 4.32	197.6 ± 5.54	Not significant
Threonine	286.56 ± 5.98	198.65 ± 7.28	Significant
Methionine	42.64 ± 5.62	44.32 ± 9.86	Not significant
Proline	186.32 ± 7.34	184.54 ± 6.54	Not significant
Cystine	223.65 ± 3.98	226.3 ± 12.96	Not significant
Arginine	178.65 ± 5.98	156.78 ± 8.64	Significant

**Table 7:** Correlation between glycaemic control and degree of aminoacidurias in type 2 diabetic subjects.

HbA1c level (%)	Glycaemic control grading	Degree of aminoaciduria	*R*-value	*T*-value	*p* value
< 6.5	Excellent control	0–2	0.12	1.23	0.22
6.5–7.5	Good control	3–5	0.35	2.56	0.01
7.6–8.5	Fair control	6–8	0.58	4.21	< 0.001
8.6–9.5	Poor control	9–12	0.75	6.15	< 0.001
> 9.5	Very poor control	≥ 13	0.85	8.42	< 0.0001

## Data Availability

The data supporting the findings of this study are available upon reasonable request from the corresponding author. The datasets generated and analysed during the current study are not publicly available due to privacy concerns but may be made available to researchers upon reasonable request and with appropriate permissions.
